# Case Report: Systematic endoscopic characterization of synchronous esophageal, gastric, and colorectal involvement in multisystem Langerhans cell histiocytosis

**DOI:** 10.3389/fmed.2025.1674312

**Published:** 2025-12-04

**Authors:** Xueman Wang, Quan Luo, Tiannu Ding, Bo Lian, Xintong Jiang, Jian Dong

**Affiliations:** Endoscopy Center, Shaoxing People's Hospital (The First Affiliated Hospital of Shaoxing University), Shaoxing, China

**Keywords:** langerhans cell histiocytosis, gastrointestinal endoscopy, mucosal fragmentation, synchronous involvement, adult

## Abstract

Langerhans cell histiocytosis (LCH) is a rare neoplastic disorder derived from dendritic cells, predominantly affecting pediatric populations. Adult-onset LCH involving the gastrointestinal (GI) tract is exceedingly rare, with limited endoscopic descriptions. We describe a 34-year-old male with gingival swelling and mandibular osteolysis, ultimately diagnosed with multisystem LCH. Imaging revealed pulmonary nodules and diffuse gastrointestinal involvement, confirmed through high-resolution endoscopy. Characteristic findings included barnacle-like esophageal plaques, ulcerated gastric nodules, and clustered subepithelial masses in the colon. A novel ‘mucosal fragmentation sign’ was identified during resection—abrupt mucosal detachment revealing white, fish-flesh-like tumor tissue. Histology confirmed LCH with CD1a, S100, and langerin (CD207) positivity, alongside detection of a BRAF V600E mutation. This case is the first to systematically characterize concurrent esophageal, gastric, and colonic LCH involvement in an adult. Distinctive endoscopic patterns may support early diagnosis and optimized biopsy strategies in atypical clinical scenarios.

## Introduction

Langerhans cell histiocytosis (LCH) is an uncommon neoplastic condition arising from myeloid dendritic precursors, with a strong predilection for pediatric patients (1–4). Adult-onset LCH is relatively rare, and its gastrointestinal (GI) manifestation is even more exceptional, with only sporadic case reports describing its features (5). The clinical presentation of GI involvement can be subtle or nonspecific, often mimicking other inflammatory or neoplastic conditions, leading to delayed diagnosis. We present a unique case of multisystem adult LCH with synchronous involvement of the esophagus, stomach, and colon, accompanied by comprehensive endoscopic documentation. Our findings offer novel morphological insights and introduce a distinctive intra-procedural sign that may improve diagnostic accuracy.

## Case description

A 34-year-old male presented to the Department of Stomatology with initial symptoms of gingival swelling and severe pain. Physical examination revealed swelling and erosions in the bilateral buccal mucosa. Pretreatment 3D-reconstruction imaging demonstrated severe bone destruction of the mandible. Subsequent gingival biopsies confirmed the diagnosis of Langerhans cell histiocytosis (LCH), prompting admission to the hematology department for systemic evaluation. Apart from gingival discomfort, the patient reported no other symptoms. He had a 2-year history of smoking (10 cigarettes per day). Family history was significant for hypertension in both parents, with no known genetic disorders. Vital signs were within normal limits, and systemic physical examination revealed no abnormalities. Admission laboratory tests showed an elevated erythrocyte sedimentation rate (23 mm/h, reference range 0–15 mm/h), increased complement C3 (1.95 g/L, reference range 0.79–1.62 g/L) and C4 (0.45 g/L, reference range 0.10–0.40 g/L), a mildly elevated squamous cell carcinoma antigen (SCC) level (1.97 ng/mL, reference range 0–1.5 ng/mL), and a positive fecal occult blood test (FOBT). Given the elevated SCC level and positive fecal occult blood test, the patient underwent sedated esophagogastroduodenoscopy and colonoscopy, which revealed LCH involving multiple gastrointestinal sites, including the esophagus, stomach, and colon. Endoscopic Findings.

### Oral cavity manifestations

**Figure 1:**
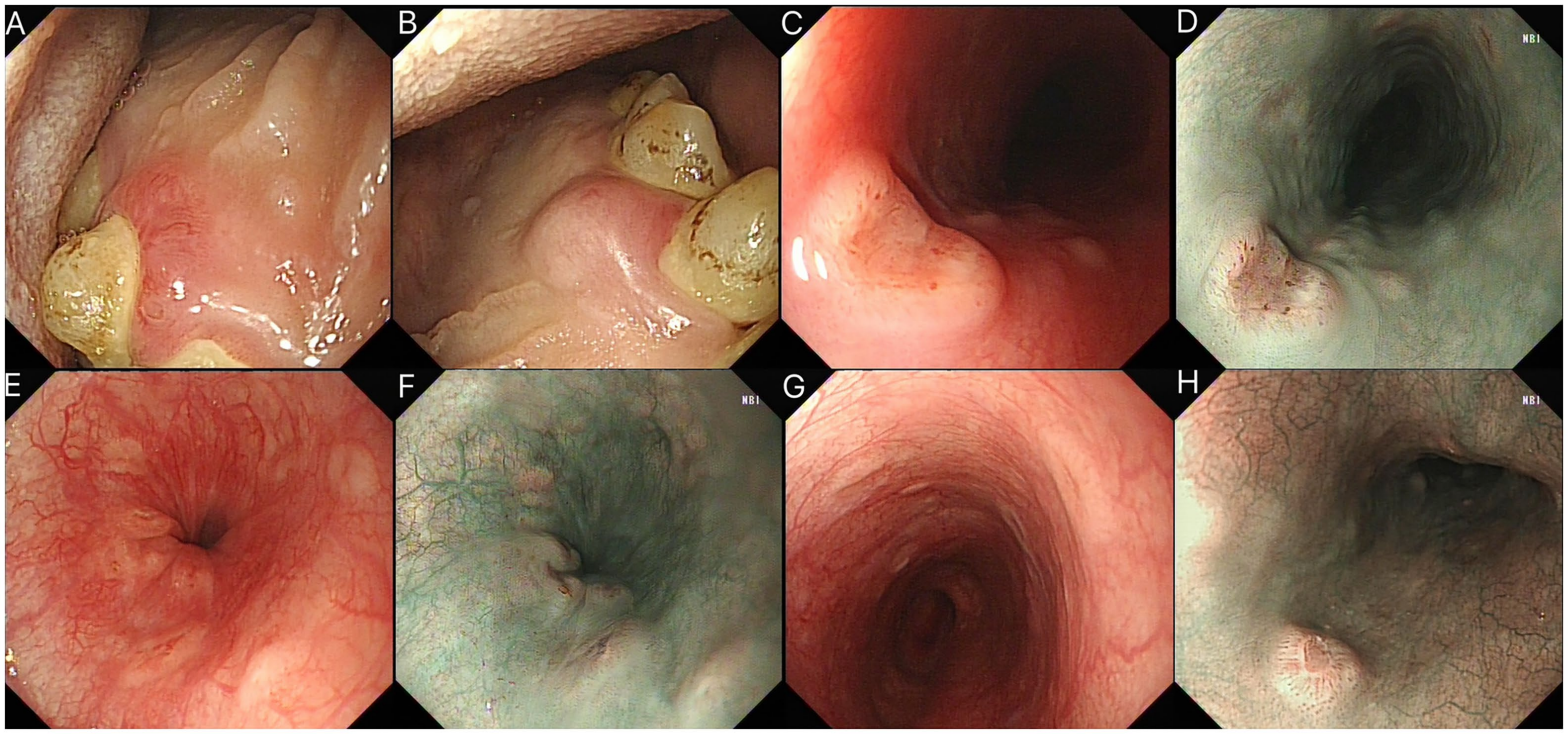
**(A,B)** Endoscopic views of oral LCH manifestations: **(A)** Left gingival erythematous swelling with telangiectatic vessels; **(B)** Right gingival SMT-like protrusion with preserved surface mucosa. **(C–H)** Endoscopic features of esophageal LCH: **(C,D)** Main lesion with central depression and radial telangiectasia; **(E–H)** Satellite barnacle-like lesions.

High-definition endoscopic imaging captured oral lesions during scope insertion. The left gingiva exhibited erythematous swelling with peripherally radiating telangiectatic vessels (Figure 1A). In contrast, the right gingiva showed a submucosal tumor (SMT)-like elevation with an intact mucosal surface (Figure 1B), suggesting heterogeneous infiltration patterns consistent with LCH.

### Esophageal involvement

Multiple round, raised esophageal lesions were identified, each featuring central depressions without exudate and sharply demarcated margins. These were accompanied by radially arranged telangiectatic vessels and smaller satellite nodules with a barnacle-like morphology (Figures 1C-H).

### Gastric lesions

**Figure 2:**
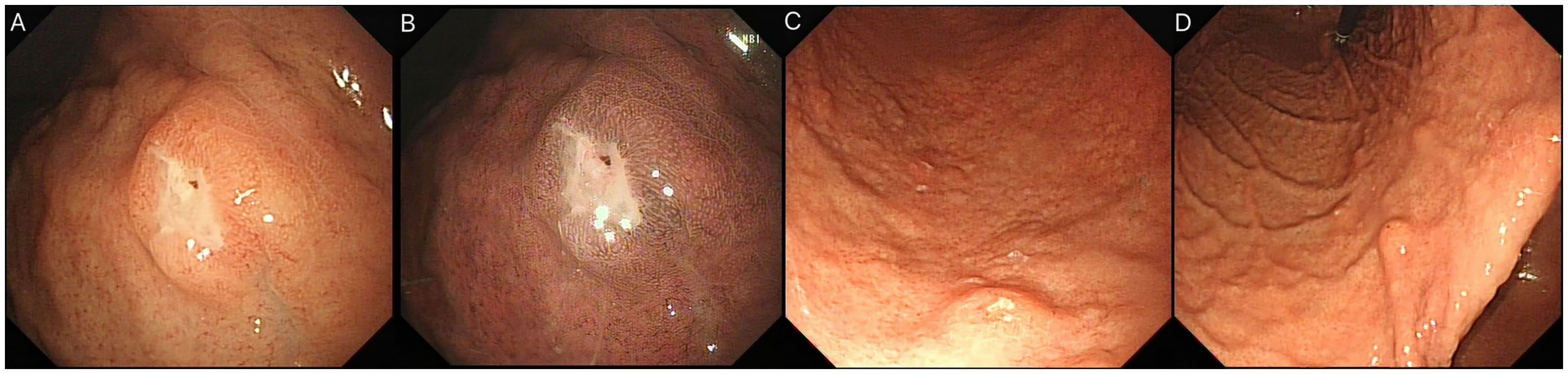
Gastric LCH features: **(A,B)** Ulcerative defect in fundus; **(C,D)** Firm submucosal protrusion with surface erosion in lower body.

Lesions in the gastric fundus appeared as distinct ulcerative defects, while the lower gastric body presented firm, biopsy-confirmed submucosal protrusions with punctate erosions (Figure 2). All sampled lesions tested positive for LCH infiltration on histopathological analysis.

### Colorectal involvement

**Figure 3:**
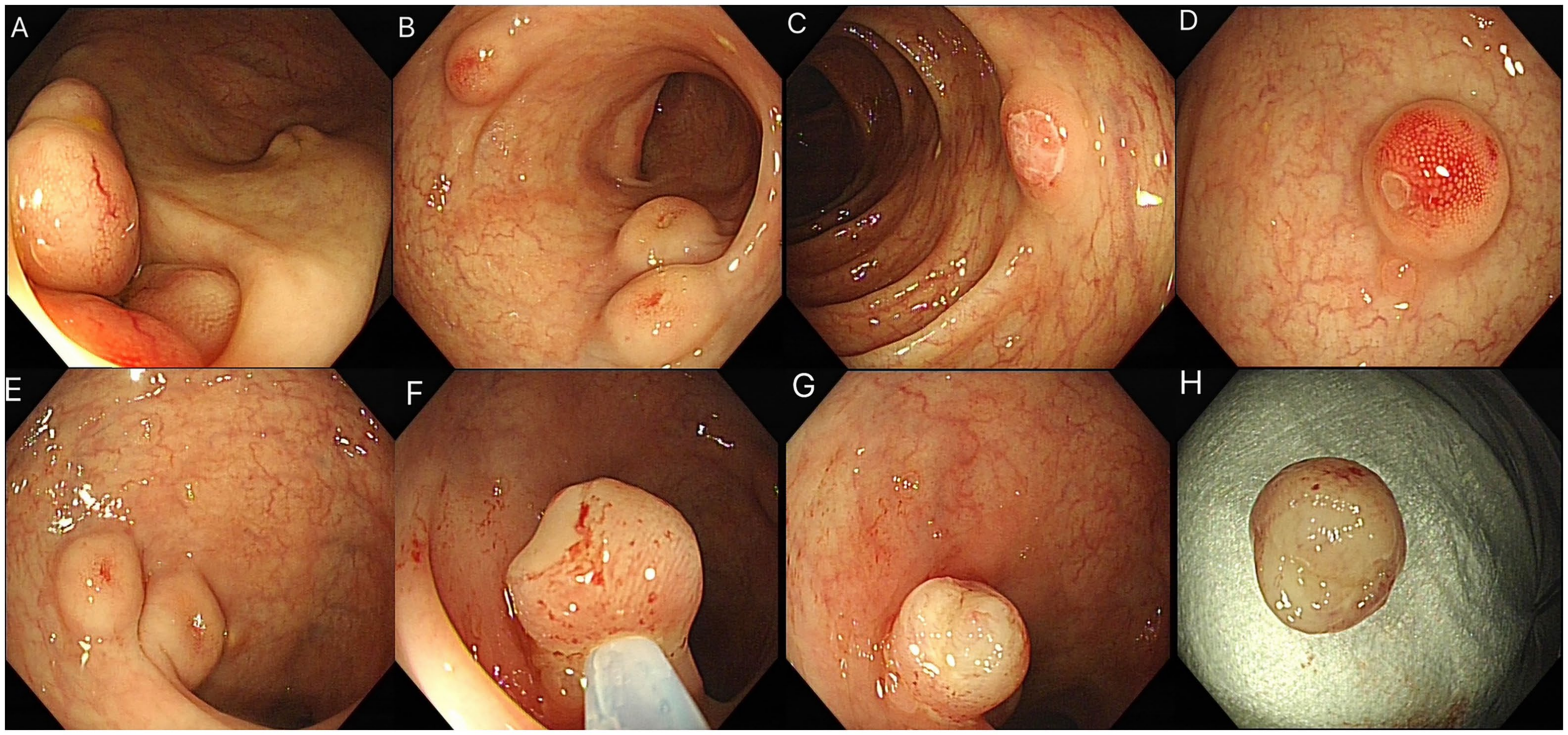
**(A–D)** Colonic LCH manifestations: **(A,B)** Cluster of submucosal protrusions in the ileocecal region and sigmoid colon; **(C,D)** Lesions with small superficial ulcerations. **(E–H)** Endoscopic resection of colonic LCH lesion: **(E)** Sigmoid colon lesion with congested mucosa; **(F)** Snare positioned at lesion base; **(G)** Mucosal fragmentation during tightening; **(H)** Exposed tumor tissue with fish-flesh morphology.

Diffuse submucosal protrusions were scattered throughout the colorectum, particularly clustered in the ileocecal region and sigmoid colon. Approximately 20% of lesions exhibited superficial ulceration, while the remaining masses were covered by intact mucosa (Figures 3A–D).

### Novel endoscopic observation: mucosal fragmentation sign

During endoscopic resection of a representative lesion, an unanticipated phenomenon occurred: when the snare was tightened at the lesion base, the overlying mucosa abruptly disintegrated, exposing homogeneous tumor tissue with a characteristic white, fish-flesh appearance (Figures 3E–H). This finding supports the hypothesis that mucosal disruption in LCH-related submucosal tumors may stem from rapid neoplastic expansion exerting outward radial forces, ultimately compromising the integrity of the mucosal barrier.

### Histopathological and molecular confirmation

**Figure 4:**
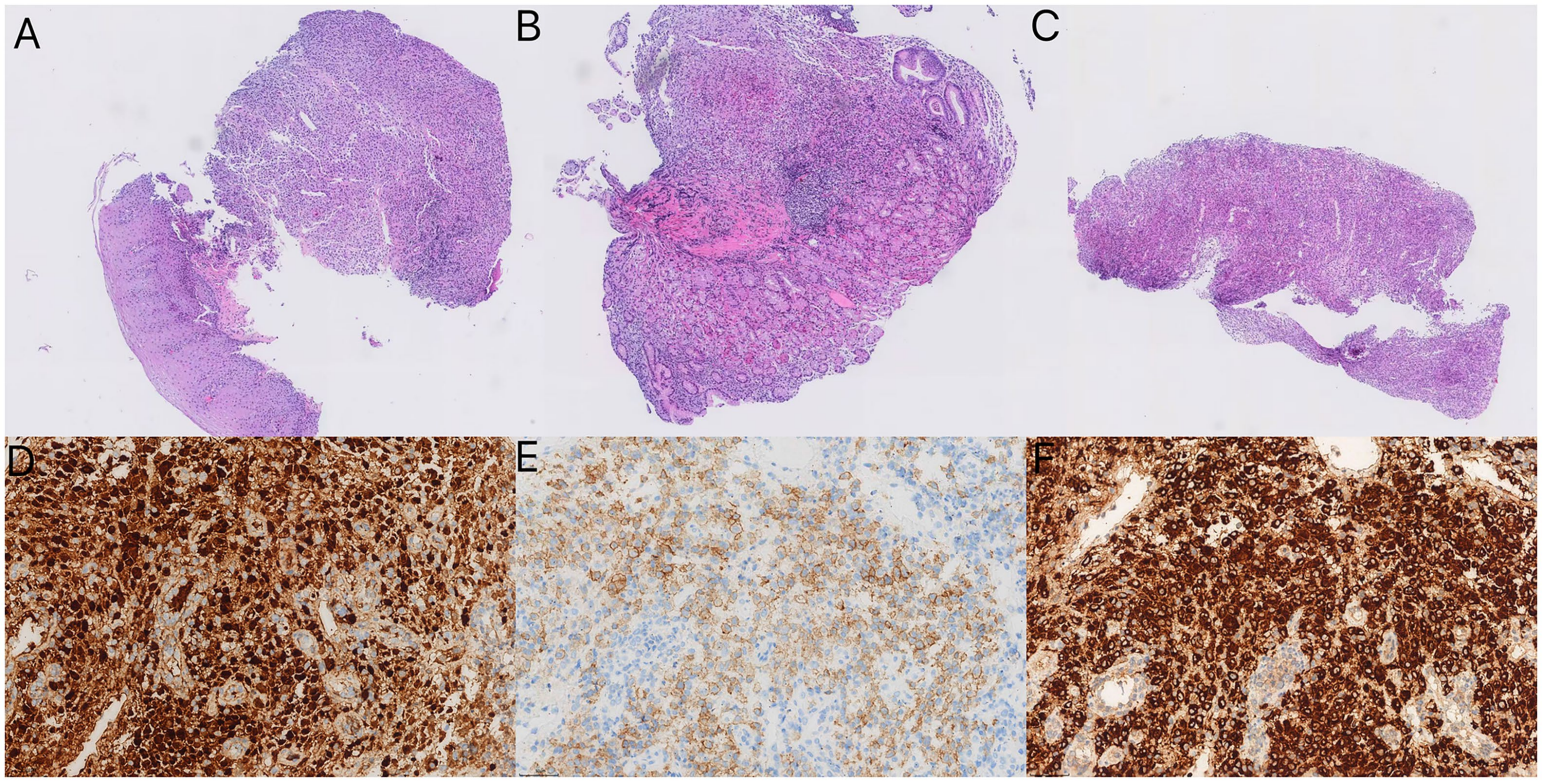
Hematoxylin and eosin (H&E)–stained sections from biopsy specimens **(A–C)**: **(A)** esophageal; **(B)** gastric; **(C)** colonic (original magnification, ×100). Immunohistochemical staining **(D–F)**: **(D)** positive for Langerin (CD207); **(E)** positive for CD1a; **(F)** positive for S100 (original magnification, ×400).

Microscopic examination revealed dense histiocytic infiltration in the esophageal, gastric, and colonic tissues (Figures 4A–C). The infiltrating cells demonstrated positivity for CD1a, S100, and langerin (CD207), confirming the diagnosis of LCH (Figures 4D–F). DNA was isolated from the colonic biopsy specimen infiltrated by Langerhans cells, and PCR analysis identified the characteristic BRAF V600E oncogenic mutation.

To further assess systemic involvement, the patient underwent a whole-body evaluation that included ^18F-fluorodeoxyglucose (FDG) positron emission tomography–computed tomography (PET/CT), contrast-enhanced abdominal magnetic resonance imaging (MRI), pituitary MRI, and bone marrow aspiration. PET/CT scan revealed multiple non-FDG-avid pulmonary nodules and cysts scattered throughout both lungs, as well as multiple hypermetabolic lymph nodes in the anterior and middle mediastinum. Notably, FDG PET/CT and abdominal MRI showed no evidence of gastrointestinal involvement. The patient was ultimately diagnosed with multisystem LCH involving the oral cavity, lungs, and gastrointestinal tract. He is currently receiving low-dose cytarabine (5-day cycles per month). This regimen was selected based on standard treatment protocols for adult LCH (6, 7). The patient exhibited a favorable clinical response to low-dose cytarabine. No dose modifications were required during the first two treatment cycles, and regular follow-up is ongoing to monitor disease status and treatment tolerance.

## Discussion

Langerhans cell histiocytosis (LCH) is a rare disorder characterized by the proliferation of Langerhans cells, a subset of dendritic cells in the immune system. Although LCH can occur at any age, it is predominantly observed in pediatric populations, with adult-onset cases being exceedingly rare. The incidence of adult LCH remains uncertain but is estimated at 1 to 1.5 cases per million individuals per year (8). The pathogenesis of LCH is primarily associated with aberrant activation of the MAPK–ERK signaling pathway, often due to mutations in genes such as BRAF (9). In adults, LCH is classified into unifocal, single-system, and multisystem subtypes (10). Multisystem involvement, particularly with gastrointestinal (GI) manifestations, is associated with a poorer prognosis.

LCH-GI is exceedingly rare. While any segment of the gastrointestinal tract can be affected, the stomach is the most frequently involved site, accounting for approximately 61.5% of cases (11). Endoscopic appearances are highly variable and non-specific, ranging from submucosal protrusions—with or without central depression or erosion—to non-specific mucosal changes such as hyperemia, erosions, ulcers, polypoid appearances, diffusely rough granular mucosa with scattered erosions, or even a normal-appearing mucosa (12). This heterogeneity significantly contributes to diagnostic delays.

To our knowledge, this is one of the first reports to provide a systematic endoscopic characterization of synchronous LCH involvement across the esophagus, stomach, and colorectum in an adult (13). This comprehensive documentation allows for the identification of several distinctive endoscopic patterns that may aid in early recognition. Our patient’s esophageal lesions, for instance, exhibited classic raised plaques with central depressions, similar to a previous description by Wang et al. (14). However, our case further demonstrated unique features, including radially arranged telangiectatic vessels and surrounding barnacle-like satellite nodules. Although the specificity of these features needs validation in larger cohorts, they serve as valuable visual clues that should raise suspicion for LCH.

Furthermore, we describe a novel endoscopic observation, which we term the “mucosal fragmentation sign.” During snare resection, the overlying mucosa abruptly disintegrated upon mechanical stress, exposing the underlying homogeneous, white, fish-flesh-like tumor tissue. This phenomenon suggests that submucosal LCH lesions may generate substantial outward radial pressure, compromising mucosal integrity and potentially explaining the spontaneous ulcerations observed in some lesions. This sign not only provides a mechanical insight into the disease but also underscores the importance of deep biopsy techniques for obtaining diagnostic tissue from such lesions.

The diagnosis of LCH is established by identifying clonal tumor proliferation, characterized by the expression of CD1a, CD207 (Langerin), and S100 proteins. Differential diagnoses for GI LCH include poorly differentiated carcinoma, lymphoma, malignant melanoma, histiocytic sarcoma, and Langerhans cell sarcoma (LCS). It is therefore essential to avoid misdiagnosing upper GI LCH as other, more common malignant tumors (15).

A significant challenge in LCH-GI is its often insidious presentation. Patients may experience non-specific symptoms like abdominal pain, diarrhea, or weight loss, but a considerable proportion, like our patient who initially lacked GI symptoms, are asymptomatic. This underscores a critical diagnostic pitfall. The study by Matsubara et al. revealed that GI involvement in adult LCH is likely under-recognized, with a prevalence as high as 37.6% in multi-organ disease, and these lesions are frequently PET-negative (16). In keeping with these observations, our case showed extensive gastrointestinal involvement that was not detected by 18F-FDG PET/CT or MRI. Several factors may account for this finding: (i) LCH lesions confined to the mucosa or submucosa and small enough to fall below the spatial resolution of current clinical imaging; (ii) low glycolytic activity in some infiltrates, yielding false-negative 18F-FDG PET results; and (iii) limited sensitivity of conventional MRI for thin mucosal disease. Additionally, our patient’s laboratory tests showed elevated SCC and a positive FOBT. Whether these biomarkers indirectly indicate LCH-GI and help identify high-risk patients for endoscopic evaluation remains uncertain and warrants validation in larger clinical cohorts. In conclusion, given the rarity and diagnostic challenge of LCH-GI, we propose that recognition of its distinctive endoscopic patterns—including multifocal submucosal protrusions, esophageal lesions with radial telangiectasia and barnacle-like satellites, and the novel “mucosal fragmentation sign”—can facilitate a timely diagnosis. For patients with confirmed LCH in any organ, proactive endoscopic evaluation should be considered, regardless of the presence of GI symptoms or negative imaging findings. Long-term follow-up remains essential to monitor treatment response and disease progression.

## Patient perspective

The patient expressed hope that the endoscopic images from his case might help other clinicians recognize LCH-related gastrointestinal manifestations more rapidly. He shared that, at first, he misattributed his symptoms to gingivitis and did not seek further evaluation. Reflecting on his experience, he hoped that the documentation of his case would support earlier diagnosis and intervention for other patients facing similar diagnostic uncertainty.

## Data Availability

The original contributions presented in the study are included in the article/supplementary material, further inquiries can be directed to the corresponding author/s.
